# Late gestation MRI to assess maternal pelvimetry, fetal biometry and placental oxygenation: a retrospective pilot study

**DOI:** 10.1186/s12884-025-08185-9

**Published:** 2025-11-28

**Authors:** Shireen Jaufuraully, Alena Uus, Jana Hutter, Simi Bansal, Bassel H. AL Wattar, Lisa Story, Dimitrios Siassakos, Anna L. David, Mary Rutherford

**Affiliations:** 1 Wellcome EPSRC Centre for Interventional and Surgical Sciences, University College London, London, UK; 2 Elizabeth Garrett Anderson Institute for Women’s Health, University College London, London, UK; 3 Reseach Department for Early Life imaging, King’s College London, London, UK; 4 Smart Imaging Lab, Radiological Institute, Friedrich-Alexander University Erlangen- Nuremberg, Nuremberg, Germany; 5 Beginnings Assisted Conception Unit, Epsom and St Helier University Hospitals, London, UK; 6 Comprehensive Clinical Trials Unit, Institute of Clinical Trials and Methodology, University College London, London, UK; 7 Department of Women and Children’s Health, King’s College London, London, UK

**Keywords:** Assisted birth, Fetal distress, Labour, Labour outcomes, Placental dysfunction, MRI, T2* relaxometry

## Abstract

**Background:**

The role of MRI pelvimetry and fetal size estimation in predicting mode of birth and risk of operative birth have been studied. However, there are no complete MRI studies that assess the maternal pelvis, fetal dimensions, and the placenta in a single protocol, in order to better inform the likelihood of operative vaginal birth or emergency caesarean section. Therefore, the aim of this pilot study was to assess the feasibility of obtaining a comprehensive prelabour assessment of maternal pelvimetry and fetal biometry using 3D MRI reconstructions, in addition to measures of placental function, in one MR examination.

**Methods:**

This was a retrospective cohort study of 29 women in late third trimester. Maternal pelvimetry and fetal measurements were performed using structural motion corrected T2 weighted MR images, placental T2* values (an indirect measure of placental oxygenation), and birth outcomes were also collected. Intra and inter-rater variability were calculated for the first 10 patients using the intraclass correlation coefficient. The correlation between manual (measuring the contour area) and calculated circumferences of maternal and fetal structures were also assessed to compare the practicability of performing the two alternative approaches.

**Results:**

People were imaged between 36 + 1 to 38 + 4 weeks’ gestation. It was possible to obtain comprehensive maternal and fetal measurements. Intra-rater variability was generally excellent, and inter-rater reliability was moderate to excellent. There was a strong correlation between manually obtained and calculated circumferences; Spearman’s ranged from 0.75 to 0.95. Placental volume, mean T2* and kurtosis were available for 23 datasets. The median placental volume was 569.7, the median T2* mean was 44.2, and the median kurtosis was 1.4.

**Conclusions:**

It is possible to perform maternal pelvimetry, fetal biometry and assess placental oxygenation from one late gestation MRI examination. The approach could be employed in a large, prospective study to ascertain whether we can predict the likelihood of assisted birth or caesarean section, with automation of image analysis to minimise inter-rater variability.

**Supplementary Information:**

The online version contains supplementary material available at 10.1186/s12884-025-08185-9.

## Background

A significant proportion of babies are born by assisted vaginal birth (AVB) and caesarean section (CS). In the UK between 2021 and 2022, 35% of women underwent CS; nearly 20% of all childbirths were by emergency CS, while 15.5% were by elective CS. 12% underwent AVB [1]. AVB rates are decreasing globally, but still account for a third of deliveries in first time mothers [2]. Critically, numbers of full dilatation CS have increased, with rates between 1.7% and 5% quoted worldwide [3, 4]. In the US, studies suggest that rates are as high as 25% [5]. Malposition and cephalopelvic disproportion (CPD)/obstructed labour are a leading cause of full dilatation CS [6].

Both AVB and CS can have serious consequences, including postpartum haemorrhage, broad ligament tears, hysterectomy [7], and of trauma to the fetus with an impacted head deep in the pelvis [8, 9]. It also carries an increased risk of scar dehiscence and preterm birth in subsequent pregnancies; a leading cause of neonatal morbidity and mortality [10]. In the UK alone, preterm delivery cost the public sector £3.4 billion over a 1-year time period [11]. Whilst there are recognised high risk groups such as primigravidae, and fetal growth restriction (FGR) there is currently no predictive tool or imaging technique to individualise the risk of second stage CS, CS for suspected fetal compromise, nor complex AVB. There are also no comprehensive imaging studies assessing factors contributing towards successful induction of labour (IOL).

Various imaging techniques and modalities have been used in attempts to predict mode of birth. Magnetic resonance imaging (MRI) is non-invasive and doesn’t rely upon ionising radiation, and hence is considered safe for mother and fetus [12]. It is complimentary to antenatal ultrasound as it can provide a large field of view of the entire uterus and maternal pelvis at late gestation, and offers excellent soft-tissue contrast. It is also superior to ultrasound in predicting neonatal birth weight in late gestation [8, 13, 14]. MRI pelvimetry has been used to identify women who are at increased risk of obstructed labour and CS, but with poor sensitivity and specificity for predicting birth outcome [15]. It is likely that prediction for birth outcomes requires a more comprehensive assessment of the fetus, placenta, maternal pelvis and uterus.

The purpose of this exploratory retrospective pilot study was to therefore determine whether it was feasible to obtain a comprehensive assessment of the maternal pelvis, fetal biometry, and the placenta, in a late third trimester MR examination, in order to explore factors that could be used in an MRI prediction model.

## Methods

A patient and public involvement group consisting of up to 30 people with personal experience of CS and AVB were consulted on the acceptability of a project such as this. Participants felt that a prospective study would be welcomed if it could change birth outcomes for the better.

### Design

Retrospective pilot study of patients undergoing late third trimester MRI.

### Ethics

Women provided written consent to one of the following ethically approved studies that were conducted at St Thomas’ Hospital, London: Placental Imaging Project (PIP, Imaging Project, REC16/LO/1573), CARdiac-Placental imaging in pregnancy (CARP, REC 19/LO/0852), Fetal Imaging with Maternal Oxygen (FIMOx, REC 17/LO/0282) and intelligent Fetal Imaging aNd Diagnosis-2 (iFind2, REC 14/LO/1806). This was in accordance with the Declaration of Helsinki.

### Patient selection

Imaging datasets from women were selected from previously conducted studies; PIP, CARP, FIMOx and iFIND2. PIP was conducted to develop MR acquisition techniques to non-invasively characterise the placenta [16]. CARP was also conducted to measure placental function, but to explore its relation to cardiac function and fetal growth in the aetiology of pre-eclampsia [17]. FIMOx assessed the maternal and fetal response to oxygen to assess placental and fetal circulation [18], and iFind2 was conducted to combine ultrasound imaging with MRI to build a fetal atlas to help computational software learn fetal anatomy [19]. Although none of the study aims at the time were to perform assessments of maternal pelvimetry or fetal biometry, nor to fully image the maternal pelvis and fetus, datasets were available where people had been imaged in late third trimester, and where the maternal pelvis, fetus, and placenta were visible. These datasets were therefore used to assess the practicality of retrospectively performing maternal pelvimetry and fetal biometry, and analysing placental data. Datasets from pregnancies with a singleton fetus imaged at over 36 weeks’ gestation, and, ideally, where the entirety of the maternal pelvis, fetus and cervix were visible on the 2D images were selected.

To assess placental volume/oxygenation in relation to gestation/outcomes, patients were excluded if there was incomplete outcome data or an absolute indication for prelabour CS such as placenta accreta.

### Image acquisition

Images were acquired using a clinical Philips 1.5T or 3 T scanners with a 24- channel torso coil or a 36-channel cardiac coil. Women were imaged either in a left lateral tilt or supine position with constant heart rate monitoring, regular blood pressure assessment and frequent verbal interaction. After an initial localizer, 2D T2 weighted Single-Shot Turbo-Spin-Echo stacks with TE = 80 or 180ms, 1.25 × 1.25 × 2.5 mm resolution and 1.25/1.5 mm slice overlap were acquired in various orientations covering the whole uterus or fetal head. The number of available stacks varied from 5 to 10. Furthermore, a multi-echo gradient echo echo-planar-imaging sequence covering the entire uterus in coronal orientation was acquired with a resolution of 2.5 mm isotropic, FOV = 360–400 × 360-400ms, 50–88 slices, TR = 8–14 s, Five TEs between 11ms and 200 ms, free breathing, TA < min.

### MRI analysis

#### Placenta

The placental multi-echo gradient-echo data was segmented by experienced observers manually masking the placental parenchyma on the data acquired using the second echo time, and carefully avoiding vasculature, maternal tissue and amniotic fluid. Datasets with visible uterine contractions were excluded. The volume as well as mean T2* and kurtosis were obtained using in-house analysis scripts [20].

#### Reconstructions of the maternal pelvis, fetus and placenta

For each of the datasets, deformable slice-to-volume registration (DSVR) [21] was used to reconstruct 3D images of the pelvis region with 1.2 mm isotropic resolution from the original motion-corrupted T2w stacks. The coverage of maternal and fetal anatomy in the output ROI varied depending on the coverage of the original stacks. In addition to motion-correction, the major advantage of DSVR reconstructed images is that they can be reformatted in any 2D plane (convenient for specific measurements). The 3D reconstructed images were then used for semi-automated 3D parcellation of the pelvis and fetal head models using a combination of manual segmentation, label propagation [22] and manual refinement. The segmentations were used to create 3D models in 3D Slicer framework [23].

#### Image analysis

**Fig. 1:**
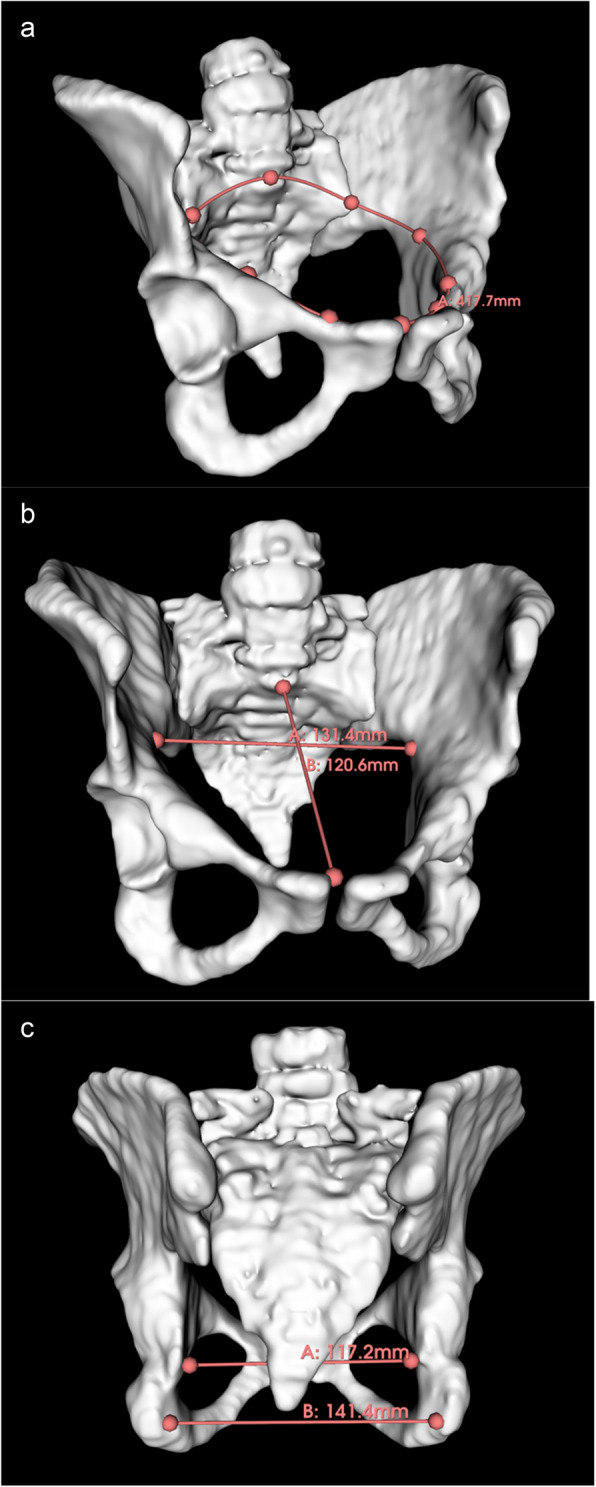
3D model of the fetal pelvis segmented from T2w MRI. (**a**) measured circumference of the inlet (**b**) transverse inlet (**A**), and the obstetric conjugate (**B**) (**c**) interspinous (**A**) and intertuberous distances (**B**).

One clinician with 6 years’ experience in Obstetrics (SJ) was blinded to clinical outcome before taking measurements of maternal or fetal structures. 3D Slicer [23], an imaging computing platform, was used to take measurements of the maternal pelvis and fetus using both 3D models and 2D reformatted planes of 3D T2w DSVR images, using the ‘markup’ module which allowed both linear and circumferential measurements to be taken in mm. Images were visually analysed to exclude any overtly abnormal anatomy or pathology. Using previously published protocols [24, 25], SJ recorded the following MRI pelvimetry values measured using 3D models: the obstetric conjugate (OC), the anteroposterior diameter (APD) and transverse diameter of the pelvic inlet, the mid pelvis APD, the circumference of the pelvic inlet and midpelvis, and intertuberous and interspinous distances (Fig. 1). Cervical length [26] and distance between femoral heads were taken using the anatomical 2D MR images in maternal sagittal and coronal planes respectively (Fig S1, Fig S2). The fetal head circumference (HC), abdominal circumference (AC) and fetal shoulder diameter were also taken using the anatomical 2D MR images (Fig S3). Measurements were achieved by using standard ultrasound landmarks [27] and previously published images/protocols [28, 29].

The correlation between both manual (based on contour area) and calculated circumferences of the fetal head circumference (HC) and abdominal circumference (AC), and inlet and midpelvis circumference of the 3D maternal pelvis was assessed to compare the two alternative approaches. To assess intra-rater variability, SJ retook the first 10 measurements. To assess interobserver variability, measurements of the obstetric conjugate, inlet APD, midpelvis APD, transverse inlet, interspinous distance, intertuberous distance, and fetal AC, biparietal diameter (BPD), occipito-frontal diameter (OFD) and shoulder width were measured by a second researcher (JB) for the first 10 patients. The formulae used to calculate the circumferences are shown below:

Pelvic inlet circumference = (inlet APD + inlet transverse) x (π/2).

Mid pelvis circumference = (midpelvis APD + interspinous diameter) x (π/2).

Fetal AC = APD x π.

HC circumference = (BPD + OFD) x (π/2).

### Clinical data collection

Maternal and neonatal outcomes were collected from Badgernet, Redcap and Windip databases. Maternal age, BMI, gestation at MRI and gestation at birth were recorded. Labour details were collected: gestation, mode of birth, and delivery indication. Birth weight, Apgar scores at birth, and cord pH were recorded.

### Statistical analysis

We reported descriptive statistics using natural frequencies and percentages. Means and ranges for pelvic/fetal measurements were calculated. SPSS software v20 was used for statistical analysis. Rater and inter-rater variability were assessed with the intraclass correlation coefficient. Spearman’s correlation coefficient was also calculated to evaluate the correlation between SJ’s manual and calculated measurements.

## Results

### Cohort

The median maternal age at booking was 34.7 years (IQR 32–36), and the median BMI was 26 (IQR 22.6–30). The median gestation at which MRI took place was 36 + 6 weeks (IQR 36 + 3 to 37 + 6). People gave birth at 39 + 2 weeks (IQR 38 + 3 to 40 + 6). The median MRI to delivery interval was 2 weeks + 6 days (IQR 1 to 3 weeks + 6 days).

### MRI analysis

**Fig. 2:**
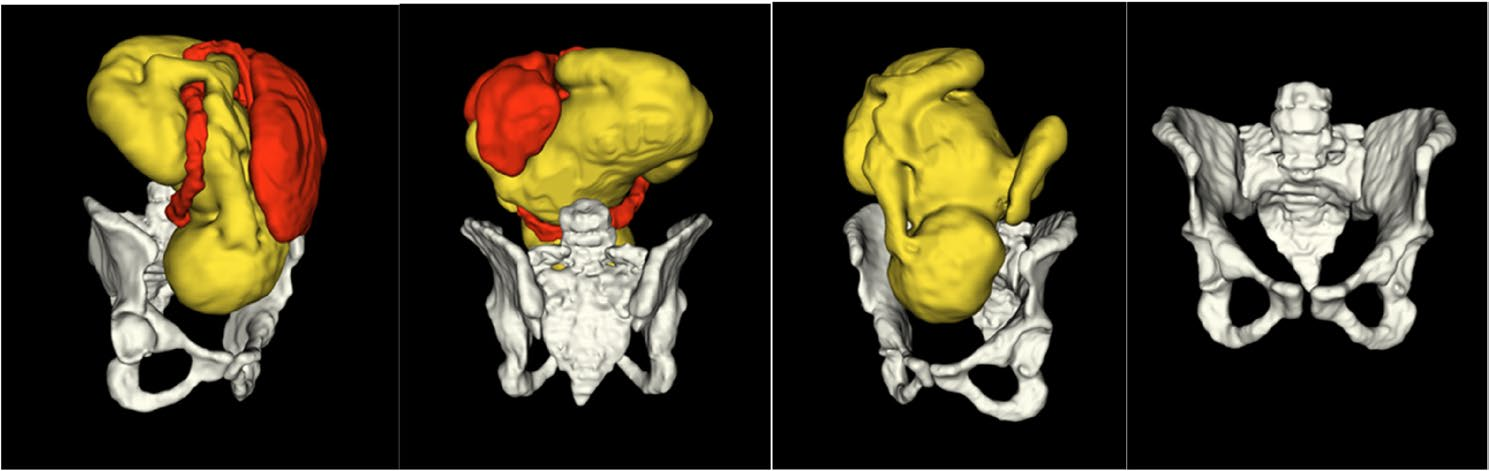
Illustration of 3D models of the segmented fetus (yellow) placenta and cord (red) and pelvis (white) derived from a 1.5T MRI dataset

At the time the four imaging studies took place, the full MRI study protocol was acquired within 60 min and was well tolerated by the participants. There were no fetal, pelvis, or placental abnormalities on visual analysis. 3D reconstructions of the head and pelvis could be produced in 17 studies. 3D reconstructions of the pelvis only were possible in 7 datasets due to incomplete ROI coverage of the entire fetus in the original T2w stacks. 3D DSVR reconstructions of the whole uterus ROI including pelvis, fetus, placenta and cord were possible in 3 patients. In 1 study, 3D reconstructions of the pelvis, fetus and cord was possible, and in another, the entire fetus and pelvis. 3D slicer allowed visualisation of the fetus, cord, placenta, and pelvis so that comprehensive visual and quantitative analysis could take place. Fetal and maternal motion in datasets was minimal because the fetuses were from the late gestational age cohort. It did not impact reconstruction quality or measurements. Three participants experienced uterine contractions during parts of the acquisition. However, this did not affect image analysis or placental T2* values, as these were derived from image stacks acquired when no contractions were present. Please refer to Fig. 2.

### Maternal measurements

It was possible to obtain all measurements detailed in Table 1 in all 29 patients. This took less than 30 min (Table 1).

**Table 1:** Means, median and ranges (mm) for measurements of the maternal pelvis

Measurement	Mean	Median (IQR)	Range
Obstetric conjugate	127	129 (122–132)	109–154
Pelvic inlet anteroposterior diameter	126	126 (121–132)	109–148
Midpelvis anteroposterior diameter	125	124 (118–131)	110–150
Transverse inlet	131	130 (127–137)	110–145
Interspinous diameter	112	111 (107–115)	101–125
Intertuberous diameter	117	118 (112–123)	92–143
Inlet circumference (measured)	411	418 (390–424)	348–441
Inlet circumference (calculated)	404	408 (390–421)	352–432
Midpelvis circumference (measured)	374	374 (360–390)	341–410
Midpelvis circumference (calculated)	373	372 (357–388)	341–413
Cervical length	33	34 (27–40)	13–47
Femoral head distance	132	133 (129–135)	109–147

### Fetal measurements

Measurements of the fetal BPD, OFD and thus calculated and manual measurements of the fetal HC, as well as shoulder diameter, were possible in all 29 babies. Due to two studies not encompassing the entire fetal abdomen, it was not possible to take a manual measurement of the circumference of the fetal AC in 2 participants, but the APD of the AC, and thus calculated circumferences, were possible for all 29 patients (Table S1, supporting information). Fetal position was clearly demonstrated in both the 3D models and 2D reformatted MR images.

### Statistical analysis

Intra-rater scores were generally excellent for both maternal and fetal measurements. There were good to excellent inter-rater scores for measurements of the maternal pelvis, with moderate to good inter-rater scores for the fetal biometry (Table S2, supporting information).

There was strong correlation between the calculated and manual measurements using Spearman’s correlation coefficient (Table S3, supporting information).

### Placental data

Placental volume, mean T2* and kurtosis was available for 23 patients. Whilst not a primary aim of the study, as clinical outcomes were available in 20 patients, the relationship between placental data and outcome was explored.

The median placental volume was 569.7mls, the median T2* mean was 44.2, and the median kurtosis was 1.4.

There was no clear relationship between maternal BMI, mode of birth, Apgar scores, or cord pH with placental data. However, there was a positive trend for higher mean T2* and neonatal birth weight (BW) (Fig S4). There was also a positive trend between higher placental volume and maternal BMI, and a negative trend between placental volume and gestational age (GA) at delivery (Fig S5a&S5b).

## Discussion

This MRI study demonstrates it is possible to acquire data to obtain maternal pelvimetry, fetal biometry and to assess placental function during one late gestation examination; essential factors influencing mode of birth. It has demonstrated that 3D MRI reconstructions can facilitate measurement of both maternal and fetal landmarks. Furthermore, fetal position or orientation did not affect the accuracy or reproducibility of measurements as 3D slicer enabled manipulation and segmentation of each image. There is good agreement between calculated and measured circumferences, indicating that both are valid ways to perform pelvimetry and fetal biometry. There was generally good to excellent intra and inter-rater variability. The study also shows some potential relationships between placental values and clinical outcomes such as fetal birthweight and gestational age at birth, but this needs to be further explored and statistically analysed in an adequately powered study. Previous studies have also demonstrated a correlation between placental T2* mean values, fetal growth restriction and preterm premature rupture of membranes [30].

One other group has prospectively generated 3D MRI reconstructions of the maternal pelvis and fetus to perform fetal biometry and pelvimetry [24] to predict mode of birth. Although our study was retrospective and smaller, the 3D pelvimetry values and fetal measurements are comparable. While there are a number of studies utilising 2D MRI [15], 3D pelvimetry Likely allows more accurate measurement of the maternal bony pelvis, as well as the ability to manipulate the pelvis into certain views, and ascertain measurements to the closest mm. Similarly, 3D reconstructions and visualisation enabled accurate 2D measures of the fetus and improved visual assessment of placental and cord characteristics.

As the sample size was small and retrospective, we have not attempted to correlate any measurements with ‘hard’ birth outcomes beyond simple measures such as birthweight and gestational age at birth. This study paves the way for a larger prospective study that incorporates at least all of the above measures with clinical outcomes, to determine whether mode of birth can be predicted, particularly for those at high risk of intervention. Such a model could include other measures not demonstrated in this study, such as fetal body volume (not attempted here as only 5 studies encompassed the fetal femur) and corresponding estimated fetal weight, and myometrial parameters not possible in this pilot because of incomplete coverage. The shape of the maternal pelvis (anthropoid, platypelloid, gynecoid, android) has not been discussed here, but has been linked to risk of AVB [31], and could also be quantified using the measures acquired.

This is a small retrospective study and as a result, not all clinical outcome data was available. In some cases, there were 2–3 weeks between the MRI scan and birth, with room therefore for ongoing changes in placental function. A prospective study might acquire more than one examination prior to labour onset. A study of MRI at onset of labour would avoid this problem but is unlikely to be feasible. Additionally, while we have drawn a potential association between placental data and some clinical data, the numbers are small, so no statistical analysis has been performed, and the outcomes are likely to be influenced by multiple factors. This too is something that can be addressed in a large prospective study.

## Conclusion

It is possible to perform a comprehensive assessment of maternal pelvimetry, fetal biometry and placental oxygenation in a single late gestation MRI, and when using 3D reconstructed images. It is clear that birth is an extremely complex process, but widely accepted that the majority of emergency births result from cephalopelvic disproportion and/or suspected fetal compromise. Provided there is standardisation and training in image analysis and measurement to optimise interrater variability, or automated tools for obtaining accurate measurements, a comprehensive prelabour MRI could and should be tested in a large, prospective study to examine whether we can better predict the likelihood of assisted birth or CS for high risk groups. Prospective studies would also need to develop a predictive tool to ascertain which clinical measures best predict mode of birth. This would then allow for individualised counselling to inform management and support informed decision making for parents and professionals.

## Supplementary Information

Supplementary Material 1.

Supplementary Material 2.

Supplementary Material 3.

Supplementary Material 4.

Supplementary Material 5.

Supplementary Material 6.

Supplementary Material 7..

Supplementary Material 8.

Supplementary Material 9.

Supplementary Material 10.

Supplementary Material 11.

Supplementary Material 12.

## Data Availability

The datasets used and/or analysed during the current study are available from the corresponding author on reasonable request.
