# Supplementary material for: Late gestation MRI to assess maternal pelvimetry, fetal biometry and placental oxygenation: a retrospective pilot study
Source: BMC Pregnancy Childbirth. 2025 Nov 28;26:14. doi: 10.1186/s12884-025-08185-9 (PMC12763823; doi:10.1186/s12884-025-08185-9)
Supplement: Supplementary file 1 — Supplementary Material 1. [file 12884_2025_8185_MOESM1_ESM.docx]

**Table S1. Means and ranges (mm) for fetal measurements**

| **Fetal Measurement** | **Mean** | **Median (IQR)** | **Range** |
| --- | --- | --- | --- |
| Biparietal diameter | 96 | 97 (94-100) | 86-103 |
| Occipitofrontal diameter | 114 | 115 (112-117) | 102-123 |
| Head circumference (calculated) | 330 | 333 (324-338) | 295-352 |
| Head circumference (measured) | 333 | 335 (322-339) | 304-359 |
| Shoulder diameter | 112 | 114 (104-123) | 81-130 |
| Abdominal circumference (calculated) | 323 | 320 (308-346) | 267-377 |
| Abdominal circumference (measured) N=27 | 333 | 325 (317-352) | 275-382 |
