# Supplementary material for: Late gestation MRI to assess maternal pelvimetry, fetal biometry and placental oxygenation: a retrospective pilot study
Source: BMC Pregnancy Childbirth. 2025 Nov 28;26:14. doi: 10.1186/s12884-025-08185-9 (PMC12763823; doi:10.1186/s12884-025-08185-9)
Supplement: Supplementary file 2 — Supplementary Material 2. [file 12884_2025_8185_MOESM2_ESM.docx]

**Table S2 intra- and inter- rater scores**

| **Maternal Measurement** | **Inter rater score** | **Intra rater score** |
| --- | --- | --- |
| Obstetric conjugate | 0.95 | 0.97 |
| APD pelvic inlet | 0.92 | 0.98 |
| APD midpelvis | 0.80 | 0.94 |
| Transverse inlet | 0.99 | 0.92 |
| Interspinous diameter | 0.79 | 0.97 |
| Intertuberous diameter | 0.77 | 0.96 |
| Inlet circumference (measured) | N/A | 0.97 |
| Inlet circumference (calculated) | 0.98 | 0.98 |
| Midpelvis circumference (measured) | N/A | 0.78 |
| Midpelvis circumference (calculated) | 0.87 | 0.92 |
| **Fetal Measurement** |  |  |
| Biparietal diameter | 0.78 | 0.92 |
| Occipitofrontal diameter | 0.54 | 0.68 |
| Head circumference (calculated) | 0.51 | 0.74 |
| Head circumference (measured) | N/A | 0.89 |
| Shoulder diameter | 0.77 | 0.96 |
| Abdominal circumference (calculated) | 0.77 | 0.92 |
| Abdominal circumference (measured) N=27 | N/A | 0.99 |
