# Supplementary material for: Late gestation MRI to assess maternal pelvimetry, fetal biometry and placental oxygenation: a retrospective pilot study
Source: BMC Pregnancy Childbirth. 2025 Nov 28;26:14. doi: 10.1186/s12884-025-08185-9 (PMC12763823; doi:10.1186/s12884-025-08185-9)
Supplement: Supplementary file 3 — Supplementary Material 3. [file 12884_2025_8185_MOESM3_ESM.docx]

**Table S3. Spearman correlation coefficient for calculated vs manual circumferences**

| **Measurement** | **Spearman correlation** |
| --- | --- |
| Inlet circumference | 0.93 |
| Midpelvis circumference | 0.75 |
| Fetal HC | 0.95 |
| Fetal AC | 0.83 |
