# Supplementary figures and images for: Late gestation MRI to assess maternal pelvimetry, fetal biometry and placental oxygenation: a retrospective pilot study
Source: BMC Pregnancy Childbirth. 2025 Nov 28;26:14. doi: 10.1186/s12884-025-08185-9 (PMC12763823; doi:10.1186/s12884-025-08185-9)

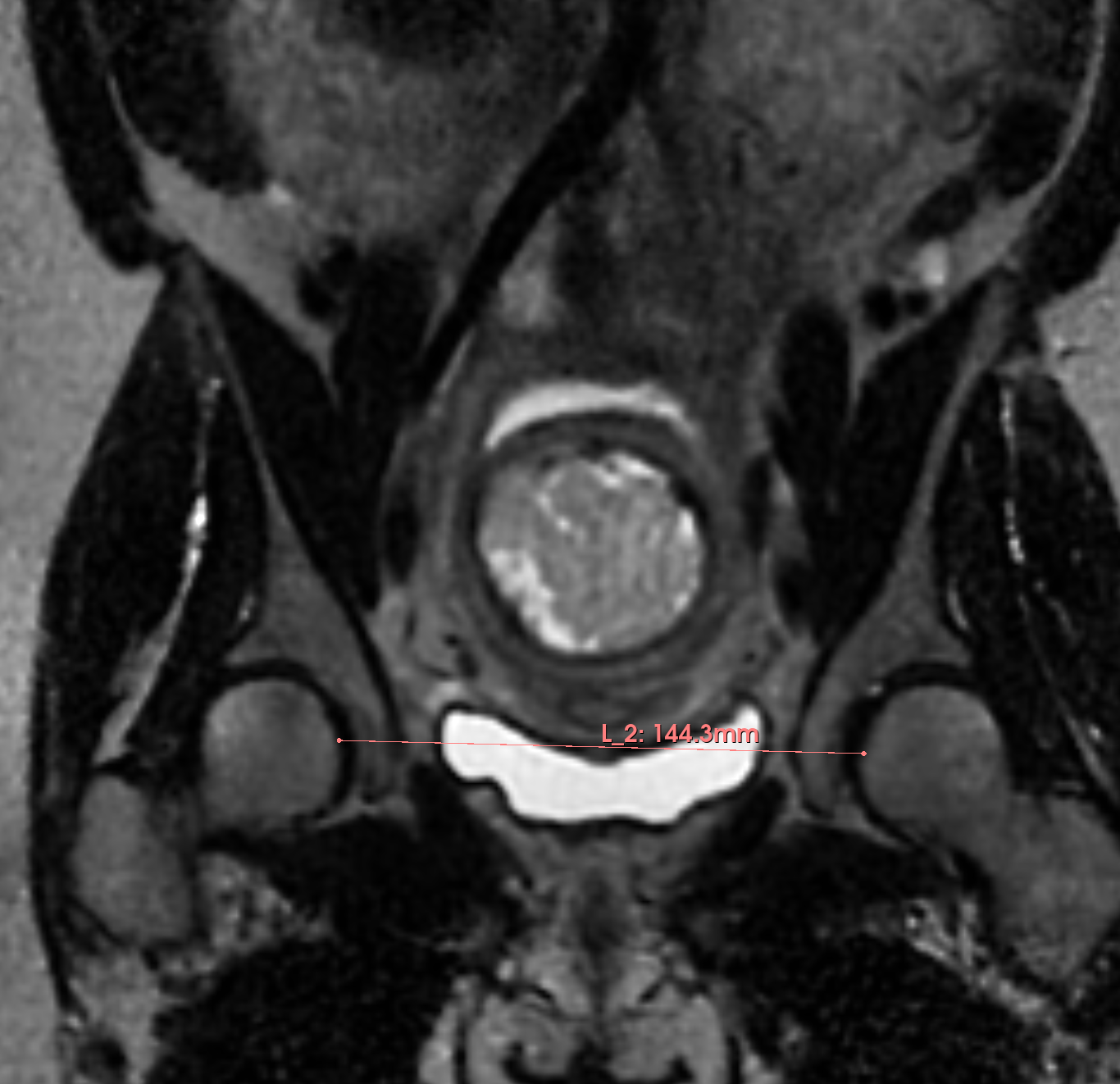

Supplement: Supplementary file 4 — Supplementary Material 4. [file 12884_2025_8185_MOESM4_ESM.tiff]

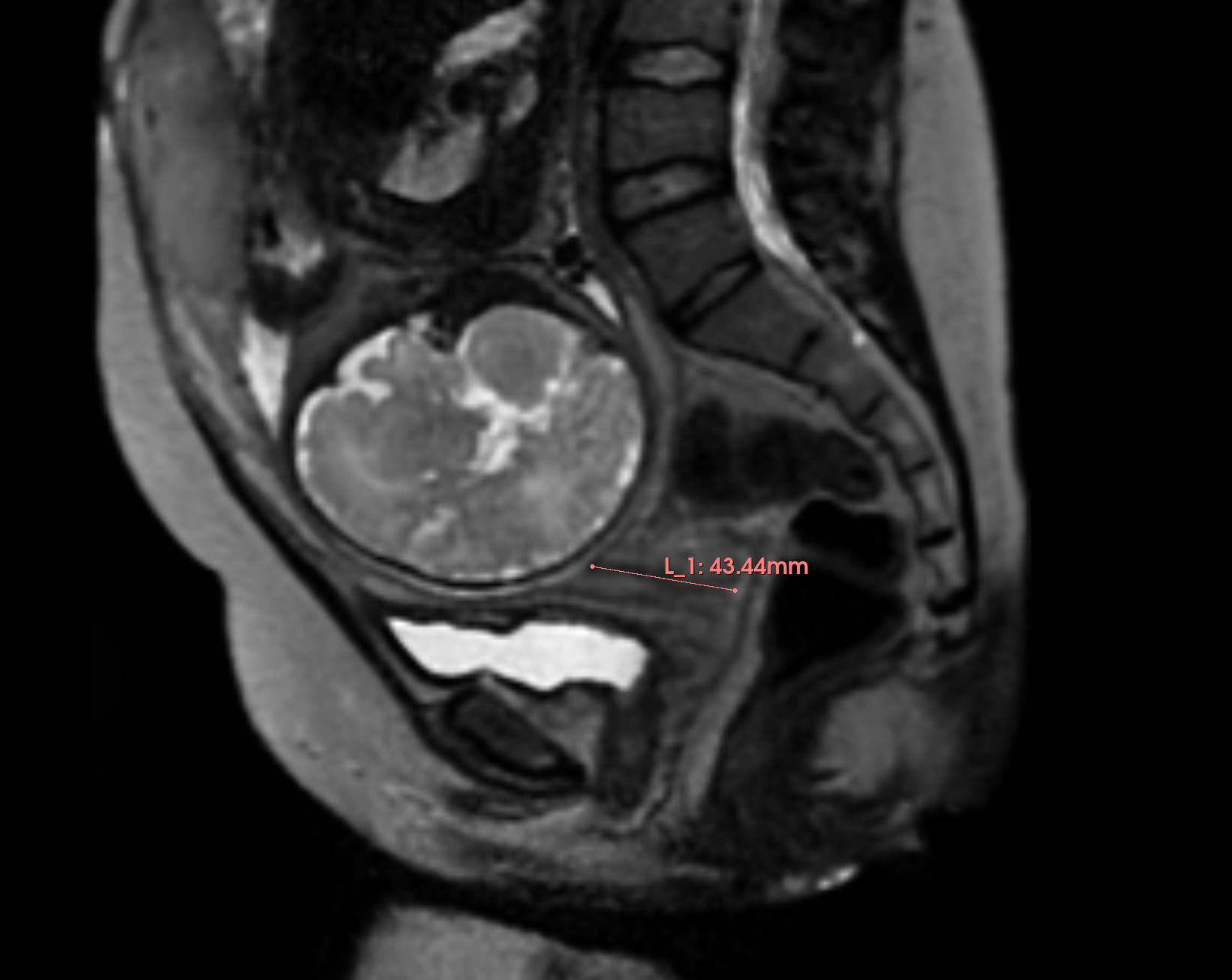

Supplement: Supplementary file 5 — Supplementary Material 5. [file 12884_2025_8185_MOESM5_ESM.tiff]

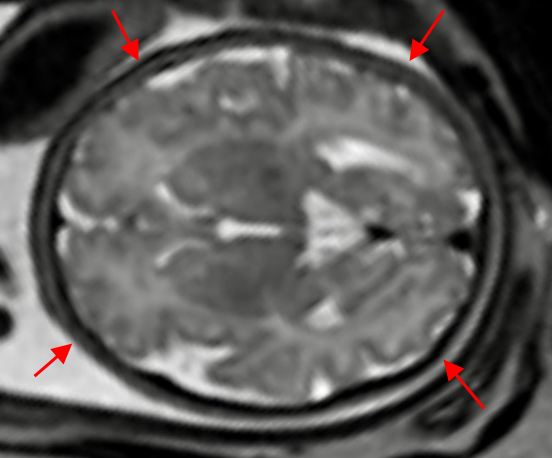

Supplement: Supplementary file 6 — Supplementary Material 6. [file 12884_2025_8185_MOESM6_ESM.tiff]

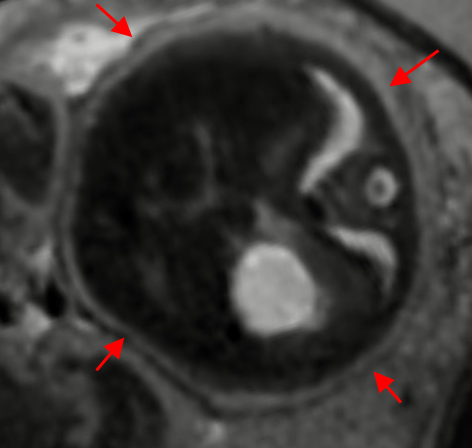

Supplement: Supplementary file 7 — Supplementary Material 7.. [file 12884_2025_8185_MOESM7_ESM.tiff]

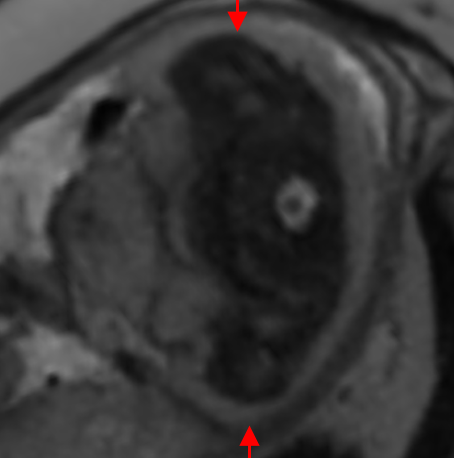

Supplement: Supplementary file 8 — Supplementary Material 8. [file 12884_2025_8185_MOESM8_ESM.tiff]
