# Supplementary material for: Late gestation MRI to assess maternal pelvimetry, fetal biometry and placental oxygenation: a retrospective pilot study
Source: BMC Pregnancy Childbirth. 2025 Nov 28;26:14. doi: 10.1186/s12884-025-08185-9 (PMC12763823; doi:10.1186/s12884-025-08185-9)
Supplement: Supplementary file 12 — Supplementary Material 12. [file 12884_2025_8185_MOESM12_ESM.docx]

**Supporting information legends:**

**Table S1.**

Title: Means, median and ranges (mm) for fetal measurements

**Table S2.**

Title: Intra- and inter- rater scores

**Table S3.**

Title: Spearman correlation coefficient for calculated vs manual circumferences

**Figure S1**

Title: Maternal coronal plane T2 weighted MRI demonstrating femoral head distance **Figure S2**

Title: Maternal sagittal plane T2 weighted MRI demonstrating cervical length

**Figure S3**

Title: Fetal biometry in reformatted 3D T2w images

Legend: a) Fetal HC, b) Fetal AC, and c) Fetal shoulder diameter. Arrows indicate where measurements should be taken

**Figure S4**

Title: Trend between placental mean T2* and neonatal birth weight

**Figure S5**

Title: Trend between placental volume and a) maternal BMI, and b) gestational age at delivery
