# Supplementary material for: Late gestation MRI to assess maternal pelvimetry, fetal biometry and placental oxygenation: a retrospective pilot study
Source: BMC Pregnancy Childbirth. 2025 Nov 28;26:14. doi: 10.1186/s12884-025-08185-9 (PMC12763823; doi:10.1186/s12884-025-08185-9)
Supplement: Supplementary file 6 [file 12884_2025_8185_MOESM10_ESM.docx]

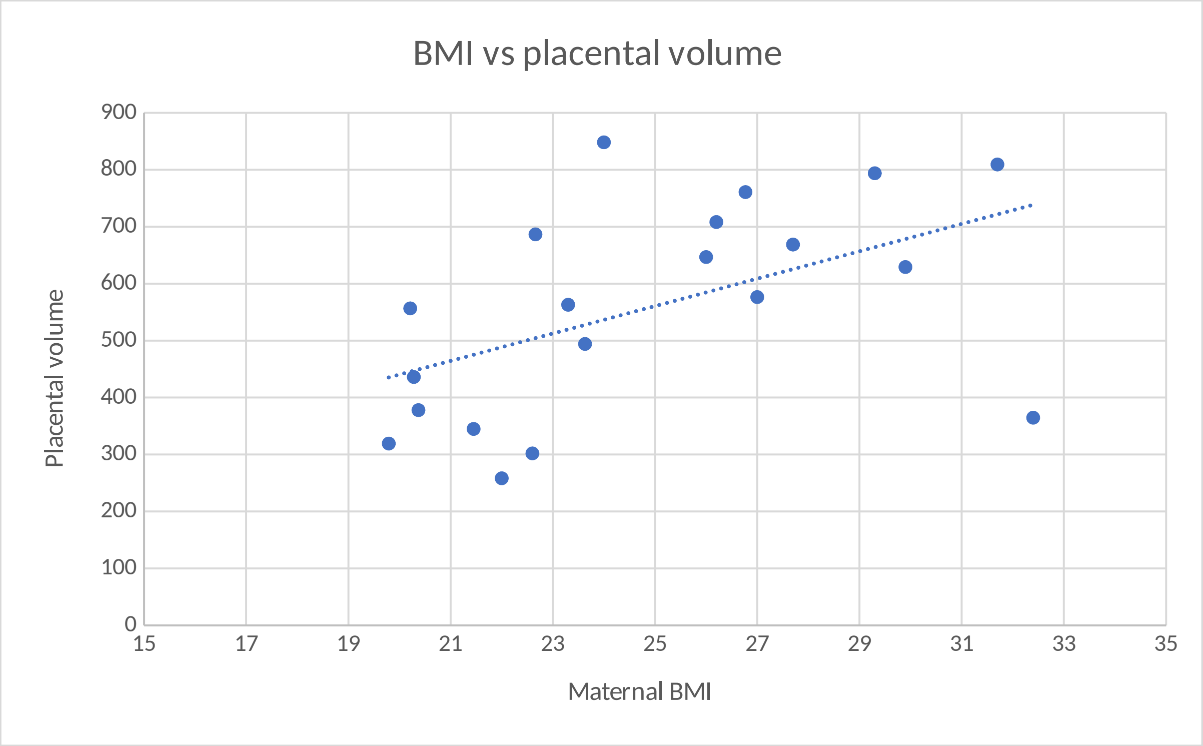
BMI vs placental volume
Placental volume
900
800
700
600
500
400
300
200
100
0
15
17
19
21
23
25
27
29
31
33
35
Maternal BMI
