# Supplementary material for: Late gestation MRI to assess maternal pelvimetry, fetal biometry and placental oxygenation: a retrospective pilot study
Source: BMC Pregnancy Childbirth. 2025 Nov 28;26:14. doi: 10.1186/s12884-025-08185-9 (PMC12763823; doi:10.1186/s12884-025-08185-9)
Supplement: Supplementary file 8 [file 12884_2025_8185_MOESM9_ESM.docx]

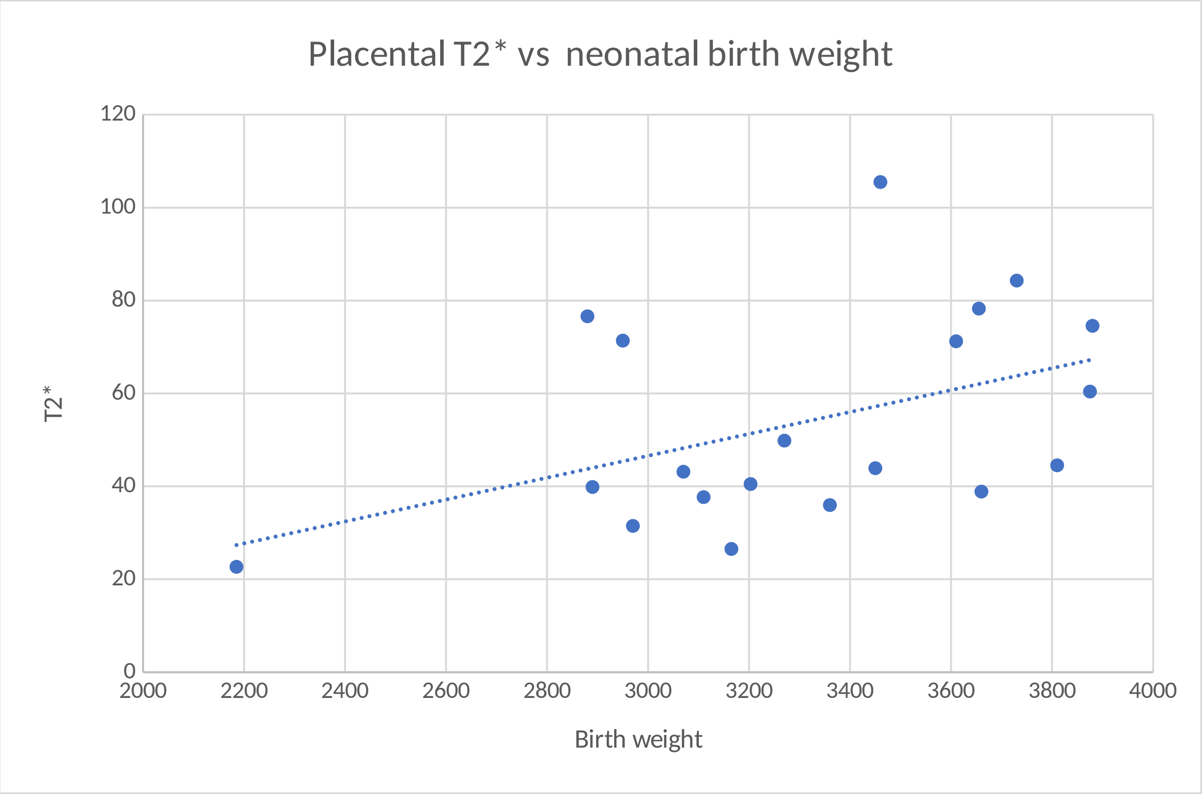
Placental T2* vs neonatal birth weight
T2*
120
100
80
60
40
20
0
2000
2200
2400
2600
2800
3000
3200
3400
3600
3800
4000
Birth weight
